# The Art of Nanoimmunobiotechnomedicine in Depression Management

**DOI:** 10.34172/apb.025.42757

**Published:** 2025-10-18

**Authors:** Dito Anurogo, Ririn Tri Ratnasari, Novi Irmania, Muhammad Sobri Maulana, Kholis Abdurachim Audah, Novi Sekar Sari, Maratu Soleha, Amir Su’udi, Ahmad Hafidul Ahkam, Andi Weri Sompa, Riswal Nafi’ Siregar, Maulida Mazaya, Riri Rimbun Anggih Chaidir, Azzah Khoridah Maulidiya, Suryani As’ad, Ami Febriza Achmad, Nur Rahmah Awaliah, Gangga Anuraga, Waode Fifin Ervina, Noorman Rinanto, Era Catur Prasetya, Tzu-Jen Kao, Tria Astika Endah Permatasari, Arli Aditya Parikesit, Nguyen Quoc Khanh Le, Nadhirah Nordin

**Affiliations:** ^1^Faculty of Medicine and Health Sciences, Universitas Muhammadiyah Makassar, Makassar, Sulawesi Selatan, Indonesia; ^2^Indonesia Molecule Institute, Malang, Jawa Timur, Indonesia; ^3^Faculty of Economics and Business, Universitas Airlangga, Surabaya, Campus B, Jl. Airlangga, Gubeng, Surabaya, East Jawa, Indonesia; ^4^Center for Halal Industry Digitalization (CHID), Surabaya, Campus B, Jl. Airlangga, Gubeng, Surabaya, East Jawa, Indonesia; ^5^Research Fellow in Faculty of Islamic Contemporary Studies, Universiti Sultan Zainal Abidin, Terengganu, Malaysia; ^6^National Research and Innovation Agency (BRIN), Jakarta Pusat, Indonesia; ^7^Poliklinik Komando Sektor III Koopsud III; ^8^Department of Biotechnology, Faculty of Health Science, Esa Unggul University, West Java, Indonesia; ^9^Sekolah Tinggi Ilmu Kesehatan IKIFA, Jakarta Timur, Indonesia; ^10^Department of Pharmacology and Clinical Pharmacy, Faculty of Pharmacy, Padjadjaran University, Sumedang, Indonesia; ^11^Teknik Informatika, Fakultas Ilmu Komputer, Universitas Pamulang, Tangerang Selatan, Indonesia; ^12^Aivita Biomedical Inc., Irvine, California, USA; ^13^Department of Biotechnology, Faculty of Life Science and Technology, Universitas Teknologi Sumbawa, Sumbawa, Indonesia; ^14^Fakultas Psikologi, Universitas Negeri Jakarta, Daerah Khusus Ibukota Jakarta, Indonesia; ^15^Department of Clinical Nutrition, Faculty of Medicine, Universitas Hasanuddin, Makassar, South Sulawesi, Indonesia; ^16^Department of Statistics, Faculty of Engineering and Science, Universitas PGRI, Adi Buana, Surabaya, East Java, Indonesia; ^17^Master of Immunology Program, Postgraduate School of Universitas Airlangga, Campus B, Jl. Airlangga, Gubeng, Surabaya, East Java, Indonesia; ^18^Electrical Engineering Department, National Taiwan University of Science and Technology, Taipei, Taiwan; ^19^Automation Engineering Study Program, Politeknik Perkapalan Negeri Surabaya, Surabaya, Jawa Timur, Indonesia; ^20^Department of Psychiatry, Faculty of Medicine, Universitas Muhammadiyah Surabaya, Jawa Timur, Indonesia; ^21^Research Center of Neuroscience, Taipei Medical University, Taipei, Taiwan; ^22^Graduate Institute of Medicine Neuroscience, College of Medical Science and Technology, Taipei Medical University, Taipei, Taiwan; ^23^International Master Program in Medical Neuroscience, College of Medical Science and Technology, Taipei Medical University, Taipei, Taiwan; ^24^Department of Nutrition, Faculty of Medicine and Health, Universitas Muhammadiyah Jakarta, Central Jakarta, Indonesia; ^25^Department of Bioinformatics, Indonesia International Institute for Life Sciences (i3L), Jakarta Timur, Indonesia; ^26^In-Service Master Program in Artificial Intelligence in Medicine, College of Medicine, Taipei Medical University, Taipei, Taiwan; ^27^AIBioMed Research Group, Taipei Medical University, Taipei, Taiwan; ^28^Faculty of Islamic Contemporary Studies (FKI), Universiti Sultan Zainal Abidin, Gong Badak Campus, Gong Badak, Kuala Nerus, Terengganu Darul Iman, Malaysia

**Keywords:** Nanoimmunobiotechnomedicine, Depression, Epigenomics, Pharmacogenomics, Neuroinflammation, Personalized medicine

## Abstract

**Purpose:**

To explore the role of nanoimmunobiotechnomedicine in depression management, emphasizing how nanotechnology and immunobiology offer innovative approaches to understanding and treating depression. We investigated the molecular mechanisms underlying depression and integrated multi-omics approaches such as genomics, epigenomics, and bioinformatics to advance therapeutic strategies.

**Methods:**

An interdisciplinary approach was applied, synthesizing data from epigenetics, nutrigenomics, and advanced bioinformatics. Furthermore, molecular and cellular neuroscience techniques were utilized alongside pharmacogenomics to deepen the understanding of depression.

**Results:**

Findings highlight the effectiveness of nano-based interventions, like targeted drug delivery systems and anti-inflammatory treatments, in reducing neuroinflammation and enhancing neuroplasticity. Multi-omics data show the importance of neurotrophic factors and gut-brain axis interactions in depression management. Additionally, pharmacogenetics suggests personalized treatment strategies, tailoring therapeutic responses based on individual genetic profiles.

**Conclusion:**

Nanoimmunobiotechnomedicine represents a frontier for personalized depression therapies. The integration of nanotechnology and immunobiology enhances bioavailability and specificity in targeting depressive disorders at the molecular level. This convergence of molecular biology, and bioinformatics studies holds significant potential to revolutionize depression treatment, offering more effective and individualized solutions for better mental health outcomes.

## Introduction

 Depression, a complex and multifaceted mental illness, is a major global public health concern arising from the interplay of various genetic, biological, psychological, and social-environmental factors. Recent scientific studies have focused on exploring the molecular mechanisms involved in the pathogenesis of depression and developing innovative approaches to diagnose and treat this disorder. According to the International Classification of Diseases, Eleventh Revision (ICD-11), depression is classified by three primary symptoms: worsening or sad mood, decreased pleasure or happiness, and depletion of energy that can lead to burnout. Other commonly associated symptoms include low self-confidence, difficulty concentrating, insecurity, feelings of culpability, pessimism, suicidal ideation, sleep disturbances, and loss of appetite. The Diagnostic and Statistical Manual of Mental Disorders, Fifth Edition (DSM-5) and ICD-11 systems are both widely used classification systems for depressive episodes. DSM-5 in particular defines a major depressive episode as the presence of five or more symptoms over a two-week period. Collectively, these classifications provide a comprehensive understanding of the intricate nature of depression and its potential impact on daily functioning.^1^

 Depression is a mental illness linked to structural alterations in the brain, particularly in the limbic region, which regulates emotions and moods. Brain imaging studies have revealed that reduced volume in the limbic area contributes to stress-related psychiatric illnesses such as depression. Additionally, functional magnetic resonance imaging (fMRI) scans have shown that mood changes induced by interleukin-6 (IL-6) are associated with heightened activity in the subgenual anterior cingulate nucleus, a brain region that plays a crucial role in mood regulation and emotional coordination and is combined with various neural circuits indicated in major depression, including the medial prefrontal cortex, amygdala, superior temporal sulcus, and nucleus accumbens.

 In addition, depression is increasingly recognized as a disorder driven by neuroinflammation, dysregulated immune signaling (notably IL-6 and tumor necrosis factor-α [TNF-α]), and impaired neuroplasticity.^2^ Immunobiology provides the mechanistic framework to understand how peripheral and central immune dysregulation—including microglial activation and kynurenine pathway induction—contributes to mood dysregulation. Nanotechnology complements this by providing precision therapeutic solutions, including nanoparticle-based delivery systems that cross the blood–brain barrier, neuroinflammation modulation, and neurotrophic support regulation (e.g., brain-derived neurotrophic factor [BDNF] signaling). These platforms can encapsulate anti-inflammatory agents, small interfering RNA, or neuroprotective molecules, thereby enhancing bioavailability, targeting specificity, and sustained therapeutic release, ultimately addressing the molecular and immunological derangements underlying depression.^3^ These showed that the neurobiological mechanisms of depression are complex and involve multiple neural circuits that influence mood, emotion, and stress response. By further investigation, researchers could gain a deeper understanding of the pathogenesis of depression and develop more effective treatments.^4^

 Nanoimmunobiotechnomedicine is an integrative biomedical approach that combines nanotechnology, immunotherapy, and biotechnology to diagnose, prevent, and treat diseases by precise modulation of immune responses at the molecular and cellular levels. This concept reflects the convergence of nanomedicine, where engineered nanomaterials enable targeted drug delivery, biosensing, and imaging, with immunobiotechnology, which leverages immune cells, cytokines, antibodies, and genetic engineering to enhance therapeutic efficacy. Such integration enables novel therapeutic modalities with improved specificity, reduced systemic toxicity, and enhanced translational potential.

 Emerging studies justify this terminology, emphasizing the synergistic use of nanomaterials in immunotherapy and biotechnology platforms to address complex diseases such as cancer, human immunodeficiency virus/acquired immunodeficiency syndrome (HIV/AIDS), and autoimmune disorders.^5^ We aimed to provide a conceptual framework that captures current translational trends in precision medicine by unifying these fields under a single term.

## Methods

 This comprehensive review employs a multidisciplinary and integrative methodology to elucidate the complex mechanisms underlying depression. It involves genetic and biological analysis to uncover the genetic, biological, psychological, and environmental determinants of depression, with a focus on epigenetic mechanisms such as deoxyribonucleic acid (DNA) methylation and histone modifications. Nutrigenomics is leveraged to understand individual dietary responses and their impact on gene expression. Molecular and cellular neuroscience is explored to investigate neurotransmitters and neuroplasticity, offering insights into therapeutic targets and personalized treatments. Epidemiological analysis is conducted to assess depression’s global prevalence and impact, while psychological and social-environmental factors are examined for their roles in depression’s development and management. A multi-omics approach integrates genomic, transcriptomic, and nutrigenomic data to provide a comprehensive understanding of depression. Additionally, pharmacogenomics and pharmacogenetics are employed to personalize treatment strategies based on individual genetic profiles.

## Epidemiology

**Figure 1:**
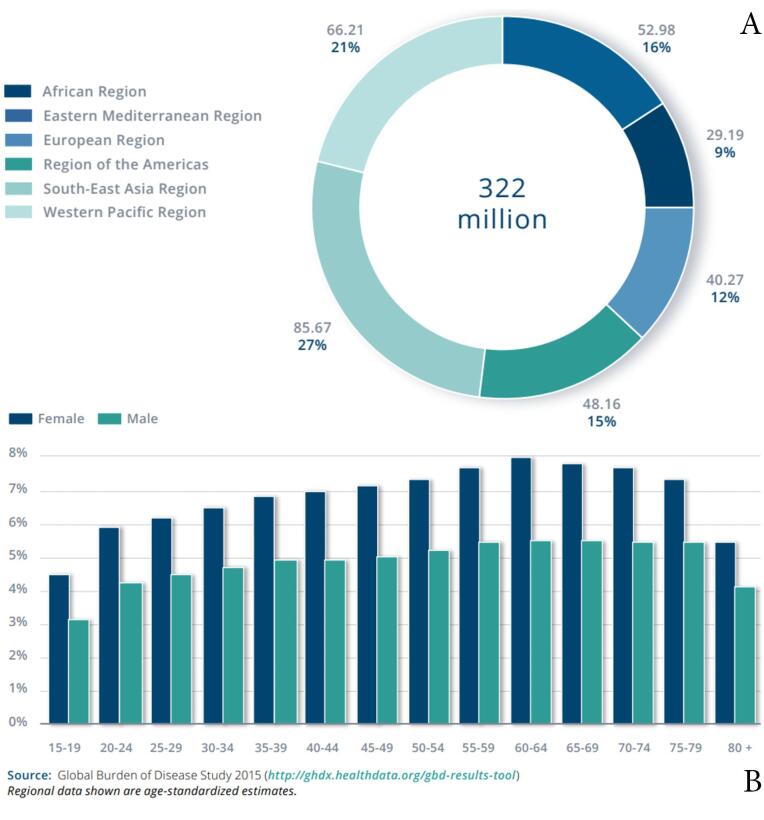
Prevalence of global depression disorders. A) Global depression burden. B) Global Prevalence of Depressive Disorders, by Age and Sex. Reference: Depression and other common disorder global health estimates, WHO, 2017. https://iris.who.int/bitstream/handle/10665/254610/WHO-MSD-MER-2017.2-eng.pdf?sequence=1

 The World Health Organization (WHO) report (2017) states that 322 million people worldwide are affected by depression. Almost 50% of these individuals reside in Southeast Asia and the Western Pacific region, indicating larger populations in these areas. (Figure 1A). The prevalence of depression varies with age, reaching its highest levels in older adults, with rates above 7.5% in females aged 55–74 years, and over 5.5% in males. Although depression is also present in children and adolescents under 15 years of age, it occurs at a lower rate than in older age groups. (Figure 1B).^6^ Based on time, the prevalence of depression increased over time. Between 2001 and 2020, the global prevalence of self-reported depressive symptoms was 34%, while major depressive disorder (MDD) affected 8% of the population. Among adolescents, the prevalence of elevated depressive symptoms has increased from 24% between 2001 and 2010 to 37% between 2011 and 2020. The highest prevalence of depressive symptoms was observed in the Middle East, Africa, and Asia.^7^

 Women universally show higher prevalence rates than men.^6-8^ Moreover, some individuals are more vulnerable to depression, including adolescents, the elderly, and those who live in regions affected by war or terrorism. For instance, prenatal depression is dangerous for birth health, and the Indian elderly population has an increased incidence of depression.^9,10^

 Depression is strongly linked to sociodemographic factors, whereby people with a low standard of living are most likely to be affected.^11^ Negative life events such as violence, natural disasters, and social inequalities increase the chances of developing depression. Depression often presents along with other chronic illnesses, including HIV/AIDS and type 2 diabetes, which makes diagnosis and management more challenging, in addition to increasing the burden on healthcare facilities.^12^ The coronavirus disease 2019 (COVID-19) outbreak is a clear example of how large-scale events affect the general well-being of individuals.^13,14^

 MDD, one of the most prevalent mental health conditions worldwide, remains under-researched and inadequately managed. Limited access to effective treatment, driven by economic constraints and social barriers, is particularly pronounced in developing countries, further contributing to the global burden.^15-17^ Culture influences how depression is treated and understood, with stigma being a factor for inadequate treatment and difficulty in healthcare access in certain nations.^18-20^

 More than 700,000 individuals die by suicide annually, making it the fourth leading cause of death among those aged 15–29. Despite the availability of effective treatments for mental disorders, over 75% of individuals in low- and middle-income countries remain untreated. The main barriers to effective care include inadequate investment in mental health services, a shortage of trained health professionals, and social stigma surrounding mental health issues.^21^

 Despite higher prevalence in low- and middle-income regions (e.g., up to 5.9% of females in the African Region according to WHO data), high-income regions such as Western Europe also exhibit substantial burdens, though often differently distributed. For example, in Western Europe, lifetime prevalence estimates range from 12 to 17%, with lower absolute suicide rates but higher chronicity and treatment uptake than in African regions.^22^ Contributing factors differ: in Africa, structural barriers (poverty, conflict exposure, stigma, and limited psychiatric infrastructure) amplify risk and under-treatment, while in Western Europe, depression is more tightly linked to social isolation, occupational stress, aging populations, and comorbid chronic diseases, despite greater healthcare accessibility. These contrasts underscore that sociodemographic determinants operate contextually, necessitating tailored public health interventions.^23^

## Depression from a psychological perspective

 Several key psychological aspects can further enrich the analysis, as summarized in Table 1. Attachment theory highlights how insecure attachment increases vulnerability through strained relationships and heightened stress responses.^24^ Cognitive distortions such as black-and-white thinking and overgeneralization also contribute to depression by encouraging negative automatic thoughts and harmful beliefs, which can trigger and prolong depressive symptoms.^25,26^ From a psychodynamic viewpoint, unconscious processes such as unresolved conflicts and buried emotions can fuel depression, with defense mechanisms like denial and projection managing distress in the short term, but worsening outcomes if overused.^27,28^

**Table 1:** Psychological theories of depression and their key concepts

**Psychological Theory/Perspective**	**Key Concepts**	**Contribution to Depression**	**Key Sources**
Attachment Theory	Early attachment styles (secure, insecure, anxious, avoidant)	Insecure attachment leads to vulnerability to depression due to disrupted caregiver relationships and increased stress responses	Spruit et al.^29^
Cognitive Theory	Cognitive distortions (all-or-nothing thinking, overgeneralization)	Distorted thinking patterns lead to negative automatic thoughts and maladaptive core beliefs, triggering and maintaining depressive symptoms	Carneiro et al.^25^; Rnic et al.^26^
Psychodynamic Theory	Unconscious processes (internal conflicts, repressed emotions)	Unresolved conflicts and repressed emotions contribute to depression, with defense mechanisms (denial, projection) worsening long-term outcomes	Cramer^27^; de Roten et al.^28^
Interpersonal Theory	Social support and relationships	Low perceived social support and interpersonal difficulties increase depressive symptoms, especially in middle-aged/older adults	Woods et al.^30^, Rajhans et al.^31^

 Interpersonal relationships and social support play a significant role in the onset and progression of depression. Low perceived support and difficulties in relationships can worsen symptoms, particularly in middle-aged and older adults.^30^ Isolation and poor communication patterns are linked to depressive disorders, with conflicts in relationships, especially among teenagers, further aggravating depressive symptoms.^31,32^ Therapies like interpersonal therapy have proven effective in easing depression by addressing relational problems and building healthier social connections.^24^ Strengthening social support and improving communication can help reduce the effects of interpersonal issues and lead to better mental health outcomes.

Table 2 displays interventions targeting psychological mechanisms in depression. Mindfulness-based interventions reduce depressive symptoms by cultivating acceptance of negative experiences.^33^ Acceptance and Commitment Therapy enhances psychological flexibility and reduces depression over time, demonstrating effectiveness in both group and online formats.^31,32^ Resilience-based approaches reinforce protective factors like optimism, self-efficacy, and social support, which are vital in protecting against depression, helping individuals handle stress, and preventing depression from developing.^34,35^ Collectively, these perspectives and interventions highlight the centrality of cognitive, relational, and resilience processes in understanding and managing depression.

**Table 2:** Interventions targeting psychological mechanisms in depression

**Intervention Approach**	**Therapeutic Focus**	**Example Interventions**	**Key Sources**
Mindfulness-Based Interventions (MBIs)	Acceptance of negative thoughts and sensations	MBIs, meditation training	Abel et al.^33^
Acceptance and Commitment Therapy (ACT)	Building psychological flexibility	Web-based or group ACT settings	Ferreira et al.^36^
Resilience Training	Strengthening self-efficacy, optimism, social support	Group-based resilience programs	Song et al.^34^

## Economic burden of depression

 According to the WHO, depression and anxiety cause global economic losses that exceed $1 trillion each year. They estimated the global costs associated with mental disorders at approximately $2.5 trillion in 2010, and projections suggest a 240% growth to $6 trillion by 2030. This confirms that depression and anxiety are the most common mental disorders that contribute significantly to these increased costs.^37^ The economic burden of depression is enormous, including medical costs that burden society, health systems, and insurance. Direct costs include direct medical costs, direct non-medical costs, and indirect costs such as sick leave and early retirement.^38^ Direct costs include medical expenses such as consultations, medications, and hospitalizations. Indirect costs stem from lost productivity due to absenteeism, presenteeism, and long-term disability. Studies have indicated that depression significantly reduces work capacity, leading to decreased economic output and increased dependence on social welfare systems. The indirect costs of depression often outweigh direct healthcare expenditures, particularly in high-income countries where lost productivity due to mental health conditions represents a significant portion of national economic losses. For instance, a comprehensive analysis across several regions showed that individuals with depression might experience a 35% reduction in work productivity, resulting in multi-billion dollar losses annually.^39^ Furthermore, comorbidities associated with depression, such as cardiovascular diseases and diabetes, further amplify these costs, as treating these overlapping conditions requires more complex and prolonged medical care.^40^ Overall, the financial burden of depression demands a strategic approach to public health policies to mitigate its long-term effects on both individual and societal well-being.

 This economic burden is aggravated by the chronic nature of the disease, as many patients require ongoing treatment, contributing to a prolonged financial strain on healthcare resources.^33,41^ Recent research highlights the pandemic-induced rise in depression rates, which has escalated overall healthcare expenditures and economic losses worldwide.^11^ Research by Culpepper revealed that many respondents with MDD reported lower socioeconomic status, indicated by factors such as disability, unemployment, low household income, and lack of education.^42^ Additionally, the more severe the MDD experienced, the lower their socioeconomic status. This suggests that individuals with a lower socioeconomic status are more susceptible to MDD and may experience a higher severity of MDD. In other words, the economic aspects of the cost burden of depression include greater financial impacts due to low socioeconomic status, increased health expenditures, and lost income and productivity.^42^ Overall, depression has a significant economic impact, affecting work productivity, health care costs, and overall societal burden.^43^

## Nutrigenomics and nutrigenetics of depression

 Nutrigenomics and nutrigenetics play a critical role in the pathobiology and management of depressive symptoms. Nutrigenetics involves studying how individual genetic variations influence the responses to specific dietary components, whereas nutrigenomics examines how nutrients affect gene expression and regulation. The interplay between these two fields could help identify biomarkers of depression, individualize treatment, and develop targeted interventions.

 Reportedly, a diet high in processed foods, saturated fats, and sugars can increase the risk of depression.^44^ However, diets rich in nutrients such as omega-3 fatty acids and vitamins B and D have been associated with a low risk of depression.^45^ The effectiveness of dietary interventions for depression may vary depending on an individual’s genetic makeup. For example, some individuals may have genetic variations that affect the absorption or metabolism of specific nutrients, leading to deficiencies that increase depression risk. Identifying these genetic variations and designing personalized nutritional plans can improve the treatment outcomes.

 Furthermore, nutrigenomics and nutrigenetics studies identify specific nutrients or dietary patterns that target depression-related pathways. For example, omega-3 fatty acids reportedly improve mood by reducing inflammation, regulating neurotransmitter function, and increasing neuroplasticity.^46-48^

 Similarly, other nutrients such as vitamin D, folate, and zinc may affect neurotransmitter synthesis, hypothalamic-pituitary-adrenal (HPA) axis function, and neuroinflammation.^49-51^ Understanding the specific mechanisms by which nutrients impact depression-related pathways can help identify novel treatment targets and develop personalized treatment plans. Overall, the integration of nutrigenomics and nutrigenetics in depression research has the potential to improve our understanding of the pathobiology of depression and develop personalized treatments.

## Multipathway of depression

 Depression, particularly MDD, is a multifactorial condition influenced by numerous interconnected mechanisms, including the activity of neurotrophins, which are essential for neuronal survival, growth, differentiation, and plasticity.^52^ An important mechanism underlying MDD-related synaptic and brain changes is disrupted neurotrophic support, which is supported by the “neurotrophic hypothesis of depression.”^53^ Neurogenesis and plasticity are affected by the expression of BDNF, a widely expressed glycoprotein.^54^ Treatment of MDD with antidepressants increases BDNF levels, which are altered by MDD. Multiple pathways are affected by brain-derived neurotrophic factor and its high-affinity receptor, tropomyosin receptor kinase B (BDNF-TrkB signaling), including synaptic activity, monoamine theory, and neurogenesis.^55^ This makes it a promising target for treating depression.

 MDD is a pathophysiologic disorder that involves pro-inflammatory cytokines, chemokines, and several mediators.^56^ Immune dysregulation and increased pro-inflammatory cytokine levels, e.g., 1IL-1 and TNF, are present in the brain and periphery of patients with MDD.^57^ The administration of bacterial lipopolysaccharides may activate microglia and other immune cells, causing pro-inflammatory cytokines to be created and inducing physiological changes that lead to depression-related symptoms.^58^ Furthermore, glucocorticoids have anti- and pro-inflammatory effects on the immune system, which is interconnected to the neuroendocrine system. Several studies have demonstrated that MDD is associated with inflammation.

 The metabolism/kynurenine pathway is also important in the pathogenesis of depression. Metabolomes comprise complex compounds that combine nutrients, metabolism, and gut bacteria. Metabolomes are believed to be a major factor in physiological processes and brain function.^59^ The brain can be directly affected by some metabolites, but others exert feedback in the periphery. Studies have shown that MDD is associated with alterations in the gut microbiome.^60^ Antidepressant effects are elicited by nutritional supplementation with probiotics or the Mediterranean diet. Nutritional and microbiotic effects on synaptic function are strongly linked to the kynurenine pathway, which contains tryptophan, an essential amino acid.^61^

 As the primary source of cellular energy, mitochondria are also responsible for mediating cellular signaling pathways.^62^ Results linking depression symptoms to mitochondrial disorders, transformed mitochondrial structure, and disrupted mitochondrial dynamics support the “mitochondria theory of depression.”^53^ Inflammatory signaling is triggered by mitochondrial dysfunction, which is accompanied by free radical production and oxidative stress.^63^ Mitochondrial damage can worsen, contributing to inflammatory responses as oxidative stress increases.^64^ Numerous depression-relevant pathways are interdependent with mitochondria, which support neurotransmission in several ways. An increase in pro-inflammatory cytokines is also caused by neurotrophic signaling when mitochondrial functions (e.g., distribution, mobility, and respiratory coupling) are compromised.^65^ Neuronal cell proliferation and death are dependent on mitochondrial function, indicating a role for low levels of reactive oxygen species (ROS) in neuroprotection.^66^

###  The role of serotonin

 Various depressive symptoms, such as anxiety, anorexia, depressed mood, libido loss, and disturbed sleep, are caused by serotonin dysregulation in major depression.^67^ Adrenocorticotropic-releasing hormone is released from the anterior pituitary gland when serotonin activates the corticotropin-releasing factor (CRF) pathway in the paraventricular nucleus, modulating the stress axis by escalating adrenocorticotropic hormone’s release.^68^

 Cortisol levels and serotonergic activity are directly correlated. Consequently, stress-induced cortisol increases are strongly correlated with increased serotonin turnover, resulting from the stimulation of tryptophan hydroxylase, which initiates the synthesis of serotonin from tryptophan.^69^ Cortisol levels are increased by chronic stress, in contrast to the release of serotonin, which is decreased by chronic stress. The tryptophan-kynurenine pathway participates in this process by activating tryptophan 2,3-dioxygenase (TDO).^70^

###  The role of glutamate

 As an essential component of pro-inflammatory cytokines, glucocorticoids, and nitric oxide, glutamate plays a vital role in the pathophysiology of depression. Moreover, pro-inflammatory cytokines and mediators, such as nitric oxide, increase glutamate release, thereby increasing intersynaptic glutamate concentrations and diminishing glutamate absorption. ^71^ Due to NMDA (N-methyl-d-aspartate) receptor stimulation, neurons and astrocytes are excitotoxically damaged, but BDNF production, a critical neurotrophic factor that regulates nervous system repair, is inhibited by extrasynaptic stimulation.^72^

 In depression, the neurotoxic potential of IL-1 and TNF-α is elevated, which causes activated microglia and astrocytes to create reactive oxygen and nitrogen species. Both are neurotoxic and oligodendrotoxic.^73^ In the amygdala, subgenual prefrontal cortex, hippocampus, astrocytes, oligodendroglia, and neuronal apoptosis (nervous system death) are lost as a result of these modifications. All of these brain regions contribute to the development of depressive symptoms.^74^

###  Excitotoxicity of glutamate 

 Glutamate is a primary excitatory neurotransmitter in the brain. Excess glutamate may contribute to depression and stress-induced apathy, in addition to neuronal atrophy. Both stress and glucocorticoids reduce glutamate clearance as long as glutamate release.^75^ The latter is caused by the loss of glia in the brain, which are responsible for clearing or removing synaptic glutamate. Excess glutamate can cause cellular damage and, in extreme cases, cell death, especially when combined with other genetic, environmental, and epigenetic factors, which diminish neuroprotective mechanisms (e.g., neurotrophic factor polymorphisms) or escalate nervous system susceptibility (e.g., exposure to hypoxia, ischemia, or hypoglycemia).^76^

## Neuroplasticity and neurogenesis

 One of the most significant discoveries of the twentieth century was the identification of pluripotent stem cells in the adult brain from which new neurons could be engineered and “reactivated.”^77^ Neurogenesis and neuroplasticity represent interconnected processes involving the generation and functional adaptation of neurons. This neuroplasticity is altered at the cellular level by inflammation and HPA axis dysfunction, both of which are triggered by environmental stress.

 In patients with MDD, the levels of BDNF, which is responsible for the regulation of neurogenesis, decrease. Decreased BDNF levels in patients with depression can be treated with antidepressant therapy, psychological interventions, or pharmacotherapy.^78^

 Limited neurogenesis in experimental animals prevents the action of antidepressants and induces depression-like symptoms, particularly in stressful situations. Consequently, it has been suggested that neurogenesis can improve resilience to stress, which is the foundation for the development of antidepressant clinical effects.^79^

 According to postmortem studies of depressed patients, untreated patients had neuronal granule deficits in the dentate gyrus compared to the treated and non-treated groups. When compared with the untreated group of patients with depression, and even when compared to the non-depressed group, the treated patients with depression had significantly different neuronal progenitor cells. These findings support mouse studies showing that antidepressants increase adult brain neurogenesis.^80^

## Model of depression

 Environmental bacteria, viruses, physiological or psychological stress, inflammation, trauma, and tissue injury are among the possible causes of peripheral cytokines, chemokines, and APPs. The HPA axis and endogenous glucocorticoids are activated by pro-inflammatory cytokines that block the HPA axis and suppress innate immunity.

 Stress increases catecholamine and cortisol production by activating the sympathetic nervous system (SNS) and HPA axis. Corticotropin-releasing hormone (CRH), adrenocorticotropic hormone (ACTH), cortisol, and catecholamines continuously influence the hormonal, immunological, and physiological stress responses of the peripheral and central nervous systems (CNS). Individuals with depression often have glucocorticoid receptor signaling anomalies and resistance due to elevated levels of pro-inflammatory cytokines and immunological intermediates. Therefore, depression increases the production of immunological intermediates.^81^ Hypothesize that the interactions between the central and peripheral nervous systems may enhance or suppress natural killer (NK) cells and T lymphocytes (T cells), which are prevalent in depression. The cytokines T helper type 1 (Th1), T helper type 2 **(**Th2), and regulatory T cells are altered in MDD.^81^

 The cerebrospinal fluid (CSF), meninges, and brain parenchyma contain immune cells that induce immune responses. MDD has been attributed to a deficiency in NK cell function, and a combination of decreased NK cell activity and increased serum IL-6 levels indicates both the repression and activation of innate immune responses. Recently, scientists have developed comprehensive models of depression. This model clarifies how innate and adaptive immunity collaborate through several molecules, causing depressive mood.^82^ Cytokines, diverse inflammatory cells, indoleamine-2,3-dioxygenase (IDO), microglia, NK cells, T cells, and neurotoxic and neurotrophic factors involved in cytokine-induced inflammatory processes, cause depressive mood. Symptoms of depression are triggered by all these factors.^83^ IL-1, IL-6, TNF-alpha, and C-reactive Protein (CRP) may contribute to the progression and onset of several psychiatric diseases, including depression. Depressed patients have cell-mediated adaptive immunity and enhanced innate immunity.^84^ As shown by the increase in the CD4 + /CD8 + T cell ratio, CD4 + T cells increased, while CD8 + T suppressor cells decreased. In addition to the activation markers CD2 + CD25 + , CD3 + CD25 + , and Human Leukocyte Antigen – DR isotype (HLA-DR + ), elevated blood levels of Interleukin-2 Receptor (IL-2R), and more B lymphocytes (B cells), people with MDD have more activated T cells and a higher proportion of activated T cells than B cells.^85^

## Biomarker of depression

 Depression is associated with the expression of immune and inflammatory biomarkers. These are CRP, cytokines, neopterin, erythrocyte sedimentation rate (ESR), and Tryptophan Catabolites TRYCATs. Major depression and elevated CRP levels have been strongly linked in previous meta-analyses.^86^ The meta-analysis found significantly higher levels of TNF-α and IL-6. Elevated IL-1 and IL-6 levels have been associated with depression in community and clinical population meta-analyses.^87^

 In patients with depression, plasma levels of neopterin are elevated. ESR levels were higher in patients with depression than in healthy volunteers. In depression, tryptophan (TRP) levels decrease, IDO levels increase, and neuroprotective TRYCAT and kynurenic acid (KYNA) decrease.^88^

###  TRYCATs pathway as indicators of depression

 Tryptophan Catabolites (TRYCATs) as measurable indicators of immune and metabolic dysregulation in MDD.^89^ The TRYCAT Pathway is one of the biological and immunological biomarkers of depression, among other biomarkers.^90^ The TRYCAT pathway, initiated by pro-inflammatory cytokine-driven activation of indoleamine 2,3-dioxygenase, links immune activation to neurotransmitter depletion (via serotonin reduction) and neurotoxicity (via kynurenine metabolites) (A). These biomarkers validate the inflammatory and metabolic aspects of depression and hold translational promise for early diagnosis, prognostication, and treatment monitoring.^91^

 Depression is correlated with the TRYCATs pathway, which includes the IDO, KYNA, and TRP pathways. However, this correlation does not hold true for everyone, which may indicate a link between various types or symptoms of depression. Researchers have investigated this relationship and discovered that varying degrees of contradiction exist between depression and TRYCAT analytes, depending on demographics and circumstances. Intriguingly, people who somatize, those who have attempted suicide, and depressed adolescents with melancholic symptoms have elevated IDO activity levels. The first step of the TRYCATs pathway involves the hydrolysis of tryptophan by the enzyme IDO, which is activated by pro-inflammatory cytokines (interferon-gamma [IFN-γ], TNF-α, and IL-6). TRYCATs can influence monoaminergic transmission and the central nervous system in neuroprotective or neurotoxic ways.^92^

 Relationships among severe depression, inflammatory biomarker pathways, and oxidative stress have been established. Increased levels of pro-inflammatory cytokines, such as IL-6, TNF-α, and IFN-γ, stimulate the production of TRYCATs and neopterin. This is achieved by upregulating enzymes such as IDO and GTP cyclohydrolase I (GTP-CH1). Antioxidant defense mechanisms such as superoxide dismutase (SOD) and glutathione peroxidase (GPx) enzymes influence oxidative stress. In contrast, the ESR and CRP levels serve as inflammatory markers. The production of 8-Hydroxy-2’-deoxyguanosine (OHdG), malondialdehyde (MDA), and isoprostanes may increase in response to an increase in oxidative stress. Inflammation and oxidative stress are linked to a feedback cycle that can increase or decrease the levels of other factors. Commonly, biomarkers of oxidative stress and antioxidant defense in the periphery have been evaluated in studies of depression. These include isoprostanes, OHdG, MDA, GPx, SOD, and reduced glutathione.^92^

## Nanotechnology perspectives on depression

 Nanotechnology offers promising strategies to enhance depression treatment by improving drug delivery and therapeutic efficacy. Traditional antidepressants often have a slow onset and unwanted side effects; nanocarriers address these limitations by enabling targeted delivery and better bioavailability.^93^ For example, advanced nanoparticles can cross the blood–brain barrier (BBB) more effectively, while self-immolating nanocapsules transport serotonin and catalase directly to the brain, reducing neuroinflammation.^94^

 Additionally, nanoparticles designed to neutralize reactive oxygen species mitigate oxidative stress,^95^ a key contributor to depression. Metal–organic frameworks (MOFs), polymeric nanoparticles (PNPs), and liposomes improve drug stability, facilitate controlled release, and minimize systemic toxicity. PNPs enhance antidepressant resistance to degradation, while MOFs provide high drug-loading capacity and targeted release profiles. Ligand modification enables nanocarriers to selectively deliver drugs, reducing off-target effects.^96^ Hybrid systems, such as MOFs combined with magnetic nanoparticles, further enhance precision therapy.^94^ Despite these advances, issues of biocompatibility and long-term stability still require investigation. ^97^

 Nanomedicine also addresses neuroinflammation and impaired neurogenesis in depression. Targeted nanocarrier systems deliver anti-inflammatory agents to the brain, reducing cytokines such as IL-6 and TNF-α.^94^ Similarly, nano-encapsulation of neurotrophic factors, such as BDNF, improves neuroplasticity and treatment outcomes. For instance, alginate-based quercetin nanogels enable intranasal BDNF delivery, bypassing the BBB and protecting it from oxidative degradation, showing significant antidepressant effects in animal models.^98^

 Phytocompound-coated nanoparticles promote both neurogenesis and autophagy, offering a dual-action therapeutic strategy. Other novel systems, such as black phosphorus nanosheets, counteract neuroapoptosis and neuroinflammation, improving cognitive function in disorders with overlapping mechanisms to depression.^99^ While these techniques are promising, achieving consistent therapeutic effects across patient populations remains a challenge.

 Nanoparticle-based systems that deliver microRNAs (miRNAs) offer an additional therapeutic avenue. Dysregulated miRNAs, such as miR-144-5p and miR-29a-5p, are associated with neuroinflammation and neuronal death in depression. Nanocarrier drug delivery systems enhance miRNA stability, protect them from degradation, and ensure precise delivery to affected brain regions.^94^ However, further research is needed to refine these systems and better understand the complex molecular pathways involved.^100^

 Recent innovations in nanomedicine enable individualized therapies by improving drug solubility, stability, and targeted release. Ligand-modified nanocarriers minimize systemic side effects, while integration with approaches such as deep brain stimulation (DBS) enhances precision in modulating neurotransmitter systems.^101^ Magnetoelectric nanoparticles (MENPs) combine targeted drug delivery with non-invasive brain stimulation, offering additional therapeutic potential. Additionally, Nanoformulations have demonstrated the ability to restore glutamate homeostasis and support neurogenesis, improving cognitive function in depression models. These advances require thorough safety assessment and regulatory approval before broad clinical adoption.

 Overall, nanotechnology is transforming depression therapy by enabling personalized, mechanism-targeted interventions. Despite its promise, significant challenges remain, particularly regarding safety, regulatory oversight, and translation from preclinical studies to clinical practice.^102^

## Futurology of depression: multi-omics perspectives on depression

###  Genomics in depression

 Genomic studies have revealed that depression, particularly MDD, is influenced by numerous genetic factors. Several polymorphisms in genes related to serotonin transport (SLC6A4), neuroplasticity (BDNF), and glucocorticoid receptors (NR3C1) have been identified as key contributors to depression. These genetic variations affect the neurotransmitter systems, stress response mechanisms, and brain structure, leading to mood dysregulation. Genomic approaches, including genome-wide association studies (GWAS), have provided insights into genetic susceptibility and paved the way for personalized therapeutic interventions.^103^

###  Transcriptomics and neuroinflammation

 Transcriptomic analyses have significantly contributed to our understanding of the molecular basis of neuroinflammation in depression. Increased expression of pro-inflammatory cytokines such as IL-6, TNF-α, and IL-1β is consistently observed in individuals with MDD, suggesting a link between immune dysregulation and mood disorders.

###  Epigenomics and depression

 Epigenetic modifications, including DNA methylation and histone acetylation, play significant roles in the regulation of gene expression during depression. Studies have shown that chronic stress leads to hypermethylation of stress-response genes, such as NR3C1, impairing HPA axis function. Histone modifications influence transcription of genes involved in neuroplasticity and neurotransmitter regulation. Targeting epigenetic regulators, such as histone deacetylase inhibitors, has the potential to develop novel antidepressant therapies.^104^

###  Nutrigenomics and depression

 Nutrigenomics, the study of how diet influences gene expression, sheds light on the role of nutrition in managing depression. Diets rich in omega-3 fatty acids, B vitamins, and polyphenols have been shown to modulate gene expression pathways related to inflammation and neurotransmission. For instance, omega-3 fatty acids enhance the expression of BDNF, which promotes neuroplasticity and protects against depression. Personalized dietary interventions based on genetic profiles can enhance treatment outcomes in individuals with depression.^105^

###  Microbiomics and the gut-brain axis

 The gut microbiome plays a pivotal role in depression through its interaction with the brain via the gut-brain axis. Dysbiosis, or an imbalance in the gut microbiota, has been linked to altered production of neurotransmitters and immune activation, both of which contribute to depressive symptoms. Metagenomic studies suggest that probiotics and prebiotics may improve mood by restoring the microbial balance and reducing inflammation. Moreover, microbial metabolites, such as short-chain fatty acids (SCFAs), influence the kynurenine pathway, a known contributor to depression.^61^

###  Proteomics in depression

 Proteomic analysis allows the identification of changes in protein expression associated with depression. Alterations in synaptic proteins, inflammatory markers, and neurotrophic factors have also been observed in individuals with depression. For instance, decreased levels of BDNF and increased levels of inflammatory proteins, such as CRP, have been linked to the severity of depression. Proteomic biomarkers offer a promising avenue for early diagnosis and treatment of MDD.^106^

###  Metabolomics and depression

 Metabolomics, the comprehensive analysis of metabolites, offers insights into biochemical changes associated with depression. Disruptions in metabolic pathways such as the kynurenine pathway, which degrades tryptophan into neuroactive metabolites, have been implicated in depression. Elevated levels of kynurenine and its toxic metabolites are associated with neuroinflammation and dysfunction. Metabolomic profiling can help identify metabolic imbalances in depression and guide the development of targeted therapies.^59^

 Omic technologies, including genomics, transcriptomics, epigenomics, microbiomics, proteomics, and metabolomics, have provided a comprehensive understanding of the molecular mechanisms underlying depression. These approaches enhance our knowledge of the disorder and pave the way for personalized medicine and novel therapeutic strategies for depression.

## Pharmacological agent in depression: antidepressant efficacy and drug-induced depression

 In depression, drugs can work as antidepressants or cause side effects of depression in their users.^107^ In this subheading, we discussed current drugs that have antidepressant activity or cause depression so that they can complement the previous discussion of depression from a demographic, cellular, and molecular perspective.

 First, antidepressant drugs for MDD can be classified into five groups based on their mechanism of action: selective serotonin reuptake inhibitors (SSRIs), fluoxetine, sertraline, and citalopram^108^; serotonin-norepinephrine reuptake inhibitors (SNRIs), venlafaxine and duloxetine^108^; tricyclic antidepressants (TCAs), amitriptyline, and nortriptyline^109^; monoamine oxidase inhibitors (MAOIs), phenelzine and tranylcypromine^110^; and mirtazapine.^111^

 The mechanism of action: SSRIs and SNRIs inhibit the reuptake of serotonin and norepinephrine, increasing their availability in the synaptic cleft.^108^ TCAs: Block the reuptake of norepinephrine and serotonin but also affect other neurotransmitters, leading to more side effects.^109^ MAOIs: inhibit the enzyme monoamine oxidase, which breaks down neurotransmitters like serotonin and norepinephrine. Atypical antidepressants have various mechanisms of action, such as inhibiting norepinephrine-dopamine reuptake (bupropion) or antagonizing certain serotonin receptors (mirtazapine).^111^ The pharmacological effects of one or more of these mechanisms involve the nervous system.

 Second, several groups of drugs can cause depression symptoms, and these include: Beta-blockers, commonly used for hypertension and heart conditions, drugs like propranolol sometimes lead to depressive symptoms^112^; Corticosteroids, medications such as prednisone, used for inflammatory conditions, can cause mood swings and depression^113^; Benzodiazepines, while used for anxiety and insomnia, long-term use of drugs like diazepam and alprazolam can lead to depressive symptoms.^114^ Anticonvulsants, medications like topiramate and levetiracetam, used for epilepsy, can have depression as a side effect, and hormonal treatments, oral contraceptives, and hormone replacement therapies can sometimes affect mood and lead to depression.^115^ Pre-existing mental health conditions may influence the occurrence of MDD symptoms, particularly in individuals with a history of depression or other mental health issues. In addition, the dosage and duration of certain medications, such as higher doses and longer durations, can increase the risk of depression. Furthermore, individual variability in genetic and epigenetic factors can affect an individual’s response to medication. Thus, it is important to manage, monitor, and educate patients to reduce the severity of depressive symptoms and increase their potential for recovery.

## Future application of bioinformatics analysis against depression

 These methods integrate data and knowledge from various resources, including genomics, proteomics, metabolomics, and clinical data, to build comprehensive models of the molecular and cellular processes involved in depression. The key aspects include data integration and analysis, network and pathway analysis, mathematical modeling, biomarker discovery, personalized medicine, and simulation for hypothesis testing. For example,^116^ used gene expression to study the underlying molecular mechanisms and biological processes to identify biomarkers of depression^117^ and employed network- and pathway-based methods to understand the molecular mechanisms underlying the pathogenesis of MDD. Thus, the systems biology approach allows for a more holistic understanding of depression by considering the interplay of multiple biological factors, thereby advancing our knowledge of its pathophysiology and aiding the development of more effective treatments. Moreover, future methods utilizing ‘multi-omics’ technologies are anticipated to uncover the links between depressive disorders and altered immune function. By employing a multiomics approach, it will be possible to identify the early stages of depression more accurately, potentially preventing more severe symptoms and identifying microbial factors that influence treatment outcomes. Finally, based on large-scale immunological, hormonal, and neuronal characterization and supported by personalized biological profiling, depression will be easier to treat. Several biomarkers can be identified to help detect depression in its early stages, allowing for individualized therapeutic approaches based on pharmacogenomics and pharmacogenetics.

## The immunobiological and immunoinformatics of major depressive disorder

 MDD is increasingly recognized as a condition involving neurotransmitter imbalances and significant immune dysregulation. From an immunobiological perspective, numerous studies have demonstrated elevated levels of pro-inflammatory cytokines, particularly IL-6, TNF-α, and IL-1β, in individuals with MDD.^118^ These cytokines can activate microglia, the resident immune cells of the brain, and trigger neuroinflammation, synaptic dysfunction, and impaired neurogenesis, contributing to the manifestation of depressive symptoms.^119^

 A key immunological mechanism implicated in MDD is the kynurenine pathway, in which pro-inflammatory cytokines induce the enzyme IDO. The activity of tis enzyme diverts tryptophan metabolism away from serotonin synthesis toward the production of kynurenine and its neurotoxic metabolites.^73,120^ This shift leads to reduced serotonin availability and increased neurotoxic load, thereby exacerbating neurodegeneration and impairing neuronal plasticity. Additionally, mitochondrial dysfunction linked to immune-related oxidative stress contributes to neuronal damage and reduced synaptic resilience.^65,121^ Targeted interventions aimed at restoring mitochondrial function and reducing ROS have shown therapeutic potential.^60,122^

 Simultaneously, immunoinformatics, an interdisciplinary field combining computational biology and immunology, has become a valuable approach for elucidating the complex immune mechanisms underlying depression. Computational modeling helps in identifying molecular targets within cytokine networks, predicting neuroinflammatory pathways, and mapping immune cell interactions, including alterations in natural killer (NK) cell activity, T-cell function, and Th1/Th2 balance.^83^ Moreover, immunoinformatics approaches have facilitated the investigation of glucocorticoid receptor resistance and HPA axis dysregulation, which sustain chronic immune activation in MDD.^123^

 Further insights have been gained into the gut–brain axis, where immune-mediated alterations in gut microbiota influence central nervous system function through microbial metabolites, cytokine production, and tryptophan metabolism.^60^ Additionally, bioinformatics-based studies have identified miRNAs, such as miR-144-5p, as key regulators of immune responses and inflammation in depression, highlighting novel opportunities for miRNA-based therapeutic interventions.^124^

 Collectively, advances in immunoinformatics have supported the development of personalized therapeutic strategies for MDD, including cytokine inhibitors, immune-modulating agents, and probiotics designed to restore immune homeostasis. This approach deepens our understanding of the immune contribution to MDD pathophysiology and accelerates the discovery of targeted, individualized treatments for this complex disorder through the integration of biological and computational insights.^123^

## Final integrative discussion

 Depression, particularly MDD, is a multifactorial condition resulting from complex interactions among neuroimmune, oxidative, metabolic, gut-brain, and epigenetic mechanisms. Central to its pathophysiology is immune-inflammatory dysregulation, marked by elevated levels of cytokines such as TNF-α, IL-6, and IFN-γ. These pro-inflammatory mediators activate the IDO enzyme, diverting tryptophan metabolism through the (TRYCAT) pathway and resulting in decreased serotonin synthesis and the accumulation of neurotoxic metabolites like quinolinic acid—processes strongly implicated in neuroinflammation and neurodegeneration.^125,126^

 Similarly, oxidative and nitrosative stress further contribute to neuronal dysfunction. Increased levels of malondialdehyde MDA, 8-OHdG, and impaired antioxidant enzymes such as SOD and GPx are consistently observed in patients with depression, underscoring redox imbalance as a key pathogenic factor.^127^ Altered serotonergic neurotransmission—evidenced by dysregulation of tryptophan hydroxylase (TPH1), serotonin transporters (SLC6A4), and receptor subtypes (HTR1A, HTR2A)—also disrupts mood regulation and emotional processing.

 Moreover, the gut–brain axis plays a critical role in depression, with dysbiosis and reductions in SCFAs, lactobacilli species, and enteroendocrine signaling (e.g., via RIN14B cells) influencing immune activity and neurotransmitter systems.^128^ Epigenetic regulation, including aberrant expression of miRNAs (miR-144-5p, miR-1202) and long non-coding RNAs (e.g., NEAT1, MEG3), further modulates inflammatory gene expression and neuroplasticity, contributing to individual susceptibility and symptom variability.^129^

 Altogether, these findings support a systems-level understanding of depression in which interconnected biological circuits—spanning immune signaling, oxidative stress, neurotransmission, gut microbiota, and gene regulation—synergistically drive disease onset and progression. Recognizing these molecular and cellular interactions advances our mechanistic insights into depression and lays the foundation for precision diagnostics and targeted nanoimmunobiotechnological interventions tailored to patient-specific pathophysiological profiles.

## Conclusion

 Nanoimmunobiotechnology provides a highly advanced and promising pathway for revolutionizing depression management. By leveraging nanotechnology, precise drug targeting, improved bioavailability, and reduced systemic side effects can be achieved, making nanotechnology an innovative solution for the limitations of traditional antidepressant therapies. The integration of multi-omics approaches, such as genomics, epigenomics, and metabolomics, further elucidates the mechanisms underlying depression and proffers personalized therapeutic strategies. Advancements in bioinformatics and machine learning have facilitated the identification of biomarkers and potential therapeutic targets. Future research should prioritize the development of nano-based treatments tailored to individual genetic profiles, ensuring greater efficacy and minimizing risks in treating complex psychiatric disorders, such as depression.

## Competing Interests

 No conflict of interest.

## Ethical Approval

 Not applicable.
